# Inhibitory effect of novel iron chelator, 1-(*N*-acetyl-6-aminohexyl)-3-hydroxy-2-methylpyridin-4-one (CM1) and green tea extract on growth of *Plasmodium falciparum*

**DOI:** 10.1186/s12936-015-0910-1

**Published:** 2015-09-30

**Authors:** Phitsinee Thipubon, Chairat Uthaipibull, Sumalee Kamchonwongpaisan, Wachiraporn Tipsuwan, Somdet Srichairatanakool

**Affiliations:** Department of Biochemistry, Faculty of Medicine, Chiang Mai University, 110 Inthawaroros Street, Tambol Sriphum, Amphur Muang, Chiang Mai, 50200 Thailand; National Center for Genetic Engineering and Biotechnology (BIOTEC), National Science and Technology Development Agency, Pathum Thani, 12102 Thailand; Division of Biochemistry, School of Medical Science, University of Phayao, Phayao, 56000 Thailand

**Keywords:** *Plasmodium falciparum*, Anti-malarial drug, Iron chelator, Green tea, 3-Hydroxypyrid-4-one

## Abstract

**Background:**

Iron is an essential micronutrient required by all living organisms including malaria parasites (*Plasmodium* spp.) for many biochemical reactions, especially growth and multiplication processes. Therefore, malaria parasite needs to take up the iron from outside or/and inside the parasitized red blood cells (PRBC). Iron chelators are widely used for the treatment of thalassaemia-related iron overload and also inhibit parasite growth at levels that are non-toxic to mammalian cells.

**Methods:**

Inhibitory effect of 1-(*N*-acetyl-6-aminohexyl)-3-hydroxy-2-methylpyridin-4-one (CM1) and green tea extract (GTE) on the growth of malaria parasite *Plasmodium falciparum* was compared with standard chelators including desferrioxamine (DFO), deferiprone (DFP) and deferasirox (DFX). A flow cytometric technique was used to enumerate PRBC stained with SYBR Green I fluorescent dye. The labile iron pool (LIP) was assayed using the calcein-acetoxymethyl fluorescent method.

**Results:**

The IC_50_ values of DFO, GTE, CM1, DFX and DFP against *P. falciparum* were 14.09, 21.11, 35.14, 44.71 and 58.25 µM, respectively. Importantly, CM1 was more effective in reducing LIP levels in the *P. falciparum* culture than DFP (*p* < 0.05).

**Conclusions:**

CM1 and GTE exhibit anti-malarial activity. They could interfere with uptake of exogenous iron or deplete the intracellular labile iron pool in malaria parasites, leading to inhibition of their growth.

## Background

Malaria is one of the most deadly infectious diseases and an enormous public health problem in tropical and subtropical countries. Malaria mortality rates have fallen by more than 25 % globally and by 33 % in the World Health Organization African Region since 2000, where almost every malarial induced death is caused by the highly virulent *Plasmodium falciparum*. Although anti-malarial drugs are used for treatment of infected patients, resistance to chloroquine (CQ), pyrimethamine (PYR) and sulfadoxine (S) drug is a major public health problem that obstructs the control of malaria [1]. Genetic polymorphisms described in *Plasmodium* spp. can provide reliable data about the prevalence of drug resistance [2]. Resistance to PYR is primarily conferred by point mutations of the *P. falciparum* genes encoded two key enzymes in folate pathway, *P. falciparum* dihydrofolate reductase (DHFR) and dihydropteroate synthase (DHPS) [3].

Plasmodial malaria parasites essentially obtain iron from their hosts for their growth and development, while the hosts have to evolve iron-withholding defence systems to suppress infection. Enhancement of iron withholding is a potential target for the development of novel therapeutic agents. Widespread multiple drug resistance in human malaria has intensified the search for new anti-malarial compounds, particularly iron chelators. The chelators exert their effects by sequestering iron from multiple sources, including transferrin as well as intracellular and extracellular iron [4]. Possibly, the iron that is bioavailable for in the intracellular parasites has originated from non-haem iron rather than the abundant haem iron in erythrocytic cytoplasm. Artemisinin found in the Chinese medicinal plant (*Artemisia annua*) binds iron to form ferric-dihydroartemisinin complex, resulting in reactive oxygen species (ROS)-mediated potent anti-malarial activity against ring and late stage of CQ-resistant *P. falciparum* malaria parasites [5].

Paradoxically, iron chelators are cytocidal to the plasmodial parasite despite the abundance of iron within the erythrocytes [6]. Interestingly, many iron chelators such as desferrioxamine (DFO), deferasirox (DFX), alkylthiocarbamates, 8-hydroxyquinoline, 2,3-dihydroxybenzoic acid (2,3-DHB) derivatives, *N*,*N′*-bis (*o*-hydroxybenzyl) ethylenediamine-*N*,*N′*-diacetic acid (HBED), *N*,*N′*-ethylenebis(*o*-hydroxyphenylglycine) (EHPG), 1-[*N*-ethoxycarbonylmethyl-pyridoxy-lidenium]-2-[2′-pyridyl] hydrazine bromide have been shown to be effective anti-malarial agents [7–13]. The compounds could possibly eliminate internal iron pools and interfere with parasite differentiation and growth, but not interfere with biological functions of red blood cells (RBC); nonetheless, some chelators may act by generating free radicals after complexing intracellular iron [14]. 3-Hydroxypyridin-4-one derivatives (e.g. CP20, CP38, CP40, CP51, CP94, and CP96) which are a family of lipophilic, orally effective bidentate iron chelators can suppress malaria in vivo and in vitro [15, 16]. Iron chelators such as DFO and DFP have been tested as chemotherapeutic agents in *P. falciparum*- or/and *Plasmodium vivax*-infected patients [13, 17, 18].

A novel orally active bidentate iron chelator named 1-(*N*-acetyl-6-aminohexyl)-3-hydroxy-2-methylpyridin-4-one or CM1 (MW = 266), a derivative of deferiprone (DFP) (MW = 139) [19], was synthesized and characterized. CM1 (K_part_ = 0.56) is more lipophilic than DFP (K_part_ = 0.11), and has a high level of affinity as well as selectivity for iron(III) ions than other metal ions [19]. It is predicted that the CM1 can readily penetrate into mammalian cells to scavenge intracellular iron, forming a neutral Fe(III)-(CM1)_3_ complex, and to efflux the neutral complex from the cells. Most importantly, CM1 is non-toxic to cultured peripheral blood mononuclear cells as well as primary mouse hepatocytes, and it can also lower levels of plasma non-transferrin bound iron (NTBI), labile plasma iron (LPI), intracellular labile iron pool (LIP) and ferritin-bound iron effectively [19–21]. Green tea extract (GTE) is comprised of polyphenolic compounds, mainly catechins, that can inhibit the iron-catalyzed generation of reactive oxygen species (ROS) [22]. Previous studies have demonstrated that GTE exhibited anti-oxidative, free radical-scavenging, iron-chelating activities in iron-loaded thalassaemic mice, and a renal-protective effect in *Plasmodium berghei*-infected mice [23, 24].

Hypothetically, iron chelators could deprive the intraerythrocytic iron essential for parasite growth or act directly on the plasmodial functional iron. Here, the inhibitory iron-chelating effects of CM1 and GTE were investigated on growth of cultured *P. falciparum*. Combined treatments of the compounds with standard anti-malarial drug were also examined.

## Methods

### Chemicals

Anti-malarial drugs, pyrimethamine (PYR, MW = 249) and dihydroartemisinin (DHA, MW = 284) were purchased from Sigma-Aldrich Company (St. Louis, MO, USA). DFO (desferrioxamine mesylate or Desferal^®^, MW = 657) was from the Novartis Pharma, Basel, Switzerland, Chiang Mai. DFP (GPO-L-One^®^ or L1, MW = 139) and DFX (Exjade^®^, MW = 373) were kindly supplied by the Institute of Research and Development, Government Pharmaceutical Organization, Bangkok, Thailand. SYBR Green I nucleic acid gel-stain (10,000× concentrate in DMSO, Cat. No. S9430) was purchased from Invitrogen, Molecular Probes (Eugene, OR, USA). Calcein acetoxymethyl (CA-AM) was purchased from Invitrogen^®^ Corporation (CA, USA). 2′,7′-Dichlorodihydrofluorescein diacetate (DCFH-DA) and epigallocatechin 3-gallate (EGCG, MW = 458) were purchased from Sigma-Aldrich Company (St. Louis, MO, USA). SYTO^®^ 61 red fluorescent nucleic acid dye (Cat. No. S11343) was purchased from Thermo Fisher Scientific Inc. (Waltham, MA, USA).

### Test compounds

#### 1-(*N*-Acetyl-6-aminohexyl)-3-hydroxy-2-methylpyridin-4-one (CM1)

The chelator was synthesized from maltol by Dr. Kanjana Pangjit at Department of Biochemistry, Faculty of Medicine, Chiang Mai University, Chiang Mai, Thailand. Protocol for CM1 production gave 20 % yield and 98 % purity. CM1’s partition coefficient constant (K_part_), equilibrium constant (pK_a_) and selectivity constant for iron(III) (pFe^3+^) values are 0.56, 3.52 ± 0.003 and 9.80 ± 0.002, and 20.3, respectively [19].

#### Green tea extract (GTE)

GTE was locally prepared from fresh tea (*Camellia sinensis*) shoots, which had been harvested from tea field in Fang District, Chiang Mai, Thailand by using the microwave method as previously described by Srichairatanakool et al. [25]. Results of HPLC analysis showed that GTE was comprised of many phytochemicals; particularly catechine derivatives of which EGCG was most abundant (24 %, w/w).

#### Drug solutions

Stock solutions (10 mM) of PYR, DHA, DFO, DFP, DFX, and CM1 were prepared in the suitable solvent, while stock GTE solution (5 %, w/v) was freshly prepared in hot water (80 ℃, 10 min). All the solutions were sterilized by passing through filter membranes (Millipore 0.45 μm, hydrophilic cellulose type). Working solutions were serially diluted in the complete medium to achieve the final concentrations ranging 0–10^−5^ M.

### *Plasmodium falciparum* culture

*Plasmodium falciparum* parasites were routinely cultured by using the Trager and Jensen method [26] with minor modifications. *P. falciparum* (strain TM4/8.2) is routinely maintained in freshly washed non-infected RBC (blood group O, Rh^−^ or Rh^+^) at 4 % haematocrit (Hct) in 10 ml of RPMI1640 medium supplemented with 25 mM 4-(2-hydroxyethyl)-1-piperazine ethanesulfonic acid (HEPES), 25 mM NaHCO_3_, 0.2 % (w/v) d-glucose, 40 mg/ml gentamicin, 50 µg/ml hypoxanthine and 10 % heat inactivated pooled normal human serum in a medium Petri dish. The culture medium comprising *P. falciparum*-infected RBC pellet (0.2 ml), non-infected RBC pellet (0.6 ml) and the supplemented RPMI1640 (9.2 ml) was incubated in 37 ^°^C incubator with 5 % CO_2_ for 48 h (one cycle of *P. falciparum*). Percentage parasitaemia was monitored on Giemsa-stained thin blood smear every 2–3 days and maintained maximally 10–15 %.

### Sorbitol synchronization of *Plasmodium falciparum*-infected RBC

*Plasmodiumfalciparum* parasites are mostly synchronized at ring stage (but not later than 10–12 h) when the sorbitol treatment is complete [27]. RBC suspension was spun down at 1800*g*, room temperature for 3 min and the supernatant was discarded. Cell pellet was resuspended in 5 % (w/v) sterile sorbitol solution (15.0 ml) and incubated at 37 ℃ for 10–15 min with shaking. After centrifugation, cell pellet containing only intraeythrocytic late ring- and early trophozoite-stage parasites was resuspended in new culture medium (10.0 ml) to obtain 4 %-Hct RBC suspension and used in experiments.

### Drug testing

#### Single drug treatment

Working drug solutions (100 µl) were dispensed in triplicate test wells and the RBC suspension (90 µl) was transferred to each well of culture plate. The plate was incubated under 5 % CO_2_ atmospheric condition for 48 h [28]. Afterwards, freshly prepared SYBR Green I solution (100 µl) was added to each well, mixed until complete haemolysis and incubated in the dark at room temperature for 1 h. Finally, fluorescent intensity (FI) (wavelength 530/575 nm) was measured by using a flow cytometer (BD Canto II) [29]. Half maximal inhibitory concentration (IC_50_) value of each drug was determined from the dose response curve representing used drug concentrations (log scale) on x axis and % parasitaemia (linear scale) on y axis. IC_50_ value represents the concentration of a compound where 50 % of its maximal inhibitory effect is observed and it is commonly used as a measure of drug’s potency.

#### Combined PYR treatment with CM1 and GTE

Various concentrations of CM1 (0–200 μM) or GTE (0–200 μM EGCG equivalent) including 30 nM PYR were dispensed into triplicate wells. Then, the RBC suspension (90 µl) was transferred to the wells and incubated under the standard condition for 48 h. Afterwards, the SYBR Green I solution (100 µl) was added and incubated for 1 h. Finally, FI was measured as described above.

### Measurement of LIP in *Plasmodium falciparum*-infected RBC

Originally, Breuer and colleagues had developed a method that used calcein, which was derived from the esterase hydrolysis of a non-fluorescent calcein acetoxymethyl ester (CA-AM), as a fluorescent probe to rapidly measure intracellular divalent metals such as Fe(II) and Cu(II) with a flow cytometer [30]. Calcein green fluorescence is quenched by the stoichiometric binding of iron (1:1), leading to a decrease in the FI signal. In this study, the modified method that utilized the calcein probe to assess the LIP of the parasitized RBC and the red fluorescent SYTO 61 dye, were used simultaneously. In the assay, parasitized RBC (5–10 % parasitaemia) were washed twice with phosphate buffered saline (PBS) containing 0.5 % Albumax II (PBS^+^) solution and inoculated into each well (2 × 10^6^ cells/well). The cells were treated with or without the test compounds (100 μM final concentrations) and incubated as described above. After the incubation period, the cells were labelled with 0.125 μM CA-AM solution for 15 min in the dark to detect the remaining intracellular iron. Then, they were labelled with SYTO 61 red fluorescent dye (5 mM in DMSO) to stain the nucleic acids of the malaria parasites, and were allowed to rest for 15 min. Finally, the FI signals (wavelength 485/530 nm for calcein; wavelength 625/645 nm for SYTO 61) of the cells were analysed through the use of a Cytek-modified FACS-Calibur flow cytometer [31].

### Detection of ROS in *Plasmodium falciparum*-infected RBC

The treated cells were labelled with non-fluorescent DCFH-DA solution (10 μM previously dissolved in methanol) for 30 min, after which the DCFH-DA would be hydrolyzed by plasma membrane esterase into reduced dichlorofluorescein (DCFH). Then, the cells were challenged with the H_2_O_2_ solution (125 μM) to convert the forming DCFH to oxidized dichlorofluorescein (DCF) and were then counterstained with the SYTO 61 fluorescent dye as described above. Finally, the FI signals (wavelength 485/530 nm for DCF; wavelength 625/645 nm for SYTO 61) of the cells were analysed through the use of a Cytek-modified FACS-Calibur flow cytometer [32].

### Statistical analysis

Data are presented as mean ± SD or/and mean ± SEM, and analysed using SPSS program (IBM SPSS Statistics 20). Statistical significance was determined by using one-way analysis of variance (ANOVA). *P* value < 0.05 is considered significant difference.

## Results

### Inhibitory effect of GTE and CM1 on *Plasmodium falciparum* growth

**Fig. 1:**
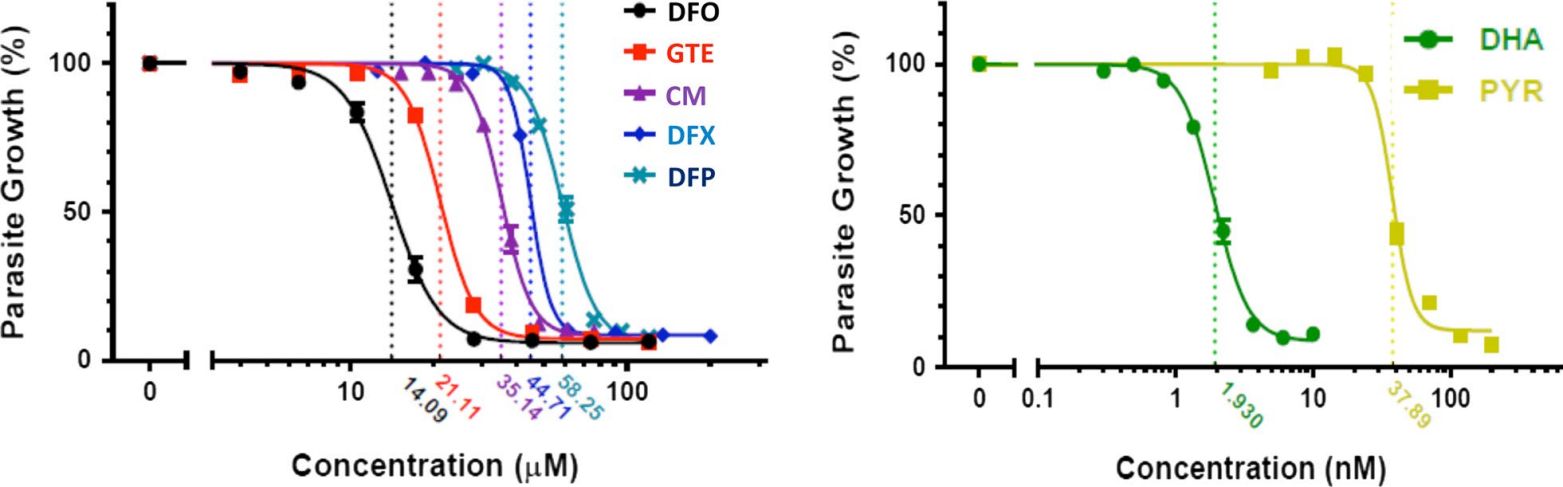
Sensitivity of *P. falciparum* to anti-malarial drugs, iron chelators and tested compounds. Data are obtained from three independent triplicate experiments and expressed as mean ± SD. Their IC_50_ values are shown in *dotted lines*

**Fig. 2:**
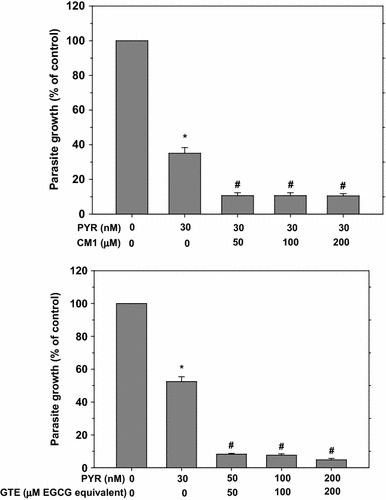
Combined effect of PYR with GTE or CM1 on growth of *P. falciparum*. Data are obtained from three independent triplicate experiments and expressed as mean ± SD. *p < 0.05 when compared with non-treatment; ^#^ *p* < 0.05 when compared with 30 nM PYR treatment

As shown in Fig. 1, DFO, GTE, CM1, DFX and DFP had different efficiencies in inhibition of *P. falciparum* growth and development as shown as % parasitaemia. Their IC_50_ values are 10.09, 21.11, 35.14, 44.71 and 58.25 μM, respectively. In comparison, DHA and PYR (IC_50_ = 1.930 and 37.89 nM, respectively) were much more potent than these compounds. In combined treatment with 30 nM PYR, CM1 (50–200 μM) significantly enhanced the anti-malarial activity of the PYR per se (mean difference of parasite growth = 24.4, 24.4 and 24.6 %, respectively). Incredibly, GTE (50–200 μM EGCG equivalent) significantly enhanced the PYR activity min a concentration-dependent manner (mean differences of parasite growth = 44.2, 44.9 and 47.7 %, respectively) (Fig. 2). Remarkably, the PYR-GTE synergy seemed to be greater than the PYR-CM1.

### Efficacy of GTE and CM1 in reduction of LIP in parasitized red blood cells (PRBC)

**Fig. 3:**
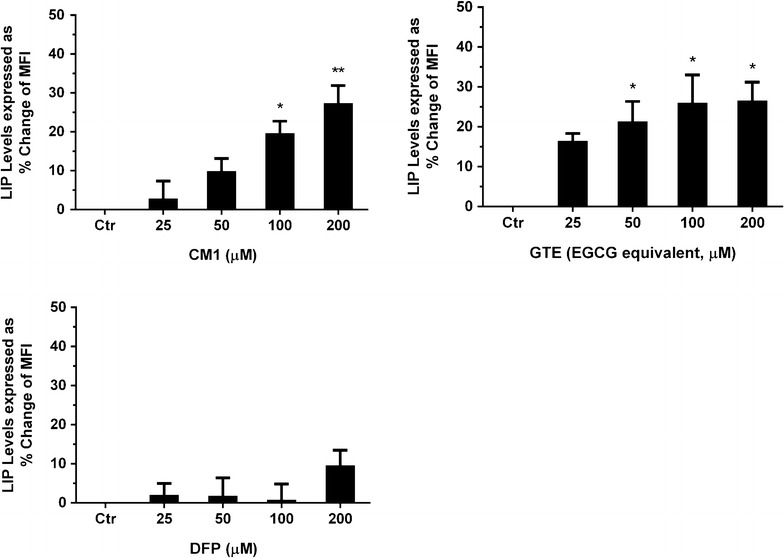
Levels of intracellular LIP in *P. falciparum*-infected RBC treated with CM1, GTE and DFP. Data are obtained from three independent triplicate experiments and expressed as mean ± SD. **p < 0.01; ***p < 0.001; ****p < 0.0001 when compared with the control

Similarly to the DFP treatment, CM1 (25–200 μM) significantly removed intracellular non-haem iron in parasitized RBC (PRBC) in a concentration-dependent manner. GTE treatments (25–100 μM EGCG equivalent doses) were also effective in removing LIP in the PRBC; nonetheless, GTE at 200 μM EGCG equivalent was not able to decrease the LIP levels any further (Fig. 3). At equivalent concentrations, CM1 and GTE would probably be more effective in reducing the LIP level in the *P. falciparum*-infected RBC than DFP.

### Free-radical scavenging activity of GTE and CM1 in PRBC

**Fig. 4:**
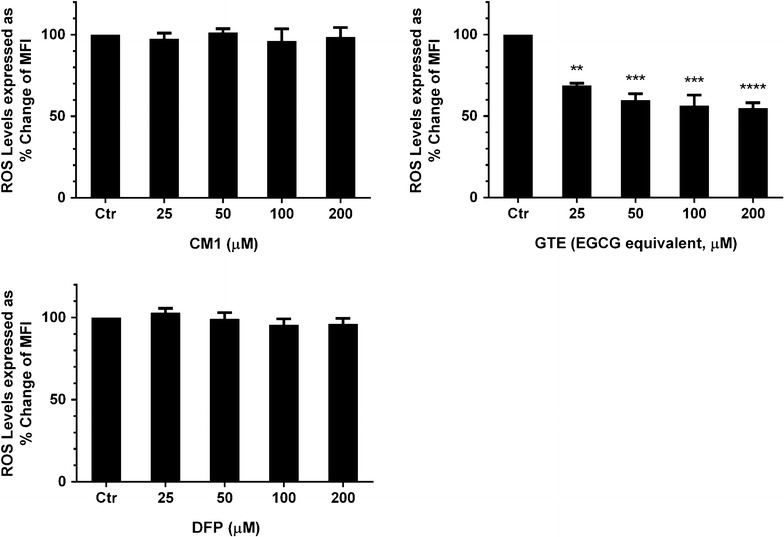
Levels of ROS in *P. falciparum*-infected RBC treated with CM1, GTE and DFP. Data are obtained from three independent triplicate experiments and expressed as mean ± SD. **p < 0.01; ***p < 0.001; ****p < 0.0001 when compared with the control

DFP and CM1 at any doses did not affect level of ROS in the PRBC. Surprisingly, GTE treatment was able to decrease the ROS level significantly in the PRBC in a concentration-dependent manner (Fig. 4). Possibly, GTE is anti-oxidative natural product that can scavenge persisting ROS and free radicals while DFP and CM1 are not.

## Discussion

Iron chelators can deplete cytosolic iron and also interact directly with functional iron in iron-containing enzymes, leading to iron deprivation of intracellular protozoa [7, 9]. In this study, GTE and CM1 showed iron-chelating property by inhibiting growth and development of *P. falciparum* at micromolar level while the reference anti-malarial drugs PYR and DHA are effective at nanomolar level. Their anti-malarial ability seems to be inversely related with their molecular size in potency of DFO > EGCG > CM1 ≃ DFX > DFP. DFO is a fungal hexadentate iron chelator used clinically for treatment of iron overload in β-thalassaemia patients, and EGCG is a natural hexadentate iron chelator used potentially for treatment of iron accumulation in Parkinson′s disease [33, 34]. Among these chelators, CM1 is the most lipophilic and more efficient than DFP in removing intracellular iron [19], possibly the compound is a more powerful anti-malarial agent. Interestingly, *N*-methylanthranilic desferrioxamine, which is a more lipophilic DFO derivative (termed reversed siderophore) can permeabilize PRBC by bypassing plasma membrane to bind intracellular iron, leading to inhibition of cultured *P. falciparum* growth (IC_50_ values = 30 ± 8 and 3 ± 1 μM, respectively) [35–37]. While losing their membrane selectivity, PRBC allow ions (e.g. Na^+^, K^+^, Zn^2+^, Fe^2+^ and Ca^2+^), polar molecules (e.g. amino acids, glucose, purine nucleosides) and even anti-malarial drugs (e.g. mefloquine, chloroquine) to pass into the cells readily [38]. By this way, influx of DFO and green tea EGCG through parasite-encoded transporters or aqueous leaks and/or pores would have occurred as well. The selectivity can be based either on the selective permeation of the chelators into the parasitized cells or on a higher susceptibility of the latter to iron deprivation or antioxidants. CM1 and DFP are orally active bidentate chelators which possess similar physicochemical and biological properties, including log b value for Fe(III) (37.0 and 37.4, respectively), pFe(III) (20.3 and 20.5, respectively), and uncharged free as well as Fe(III)-bound ligands. Extraordinarily, CM1 is more lipophilic (K_part_ = 0.53 and 0.11, respectively) and more efficient in chelating hepatic ferritin iron (approximately 18 % and 7 %, respectively) than DFO. The CM1’s log b values at pH 7.4 for Fe^3+^, Cu^2+^ and Zn^2+^ are 20.3, 18.8 and 12.9, respectively; whereas, the selectivity (pM) values for these three metal ions are 20.3, 9.8 and 6.2, respectively, suggesting higher levels in terms of the affinity and selectivity of CM1 for iron ions than other metal ions [19]. The chelator will chelate the cell irons specifically, leading to iron deprivation of the growing malaria parasites. Since malaria-infected individuals tend to be anemic or hypoeferremic, further reduction of iron stores by a non-specific chelator might be deleterious. Moreover, chelators introduced into cultures continuously for days do not quite represent the in vivo situation, as each agent has its own pharmacokinetic profile and parasite susceptibility to the chelators, as they are highly stage dependent. However, IC_50_ values of cell iron scavenging based on single time point measurement is not necessarily indicative of the chelator’s potency since the latter also depends on the permeation kinetics and pharmacokinetics. Scholl and coworkers support the evidence that malaria parasites utilize labile bioavailable iron pool(s) in red cell cytoplasm rather than haemozoin iron in the food vacuoles in order to synthesize their own haem in the mitochondria and apicomplast, while iron chelators can compete the iron utilization process and kill the parasite based on iron deprivation [6].

GTE contains polyphenols including tannic acid, EGCG, epicatechin, epigallocatechin, gallocatechin-3-gallate, and epicatechin-3-gallate. The compound can chelate irons and inhibit the Fenton reaction-mediated generation of ROS [22, 25]. GTE is indeed a natural iron chelator. Hellmann and colleagues showed that green tea EGCG inhibited *P. berghei* sporozoite motility and liver cell infection efficiently and also synergized with digitonin for these two activities [39]. ROS and oxidant drugs are toxic to biomolecules of cell components and can cause oxidative cell damage. The oxidants such as *t*-butyl hydroperoxide and artemisinin-catalyzed epoxide can damage malaria parasites and PRBC [40, 41]. Potent antioxidant GTE significantly decreased plasma levels of blood urea nitrogen and creatinine significantly in *P. berghei* ANKA-infected mice and consequently reversed their kidney function caused by oxidative stress condition [23, 42]. Controversially, EGCG and M30 (an iron chelator) did not improve survival of *P. berghei* ANKA-infected mice [43]. Similarly, hot water extract of the tea (*Artemisia annua*) containing many polyphenolic acids such as mono-caffeoylquinic acids, tri-caffeoylquinic acid, artemisinic acid, arteannuin B and rosmarinic acid exhibited synergistic anti-plasmodial activity with artemisinin in the CQ- sensitive strain of *P. falciparum* [44].

Taken together, the authors are confident CM1 and EGCG in green tea are plasmodicidal agent and function as iron-chelating agents rather than antioxidants to limit the iron supply to the malaria parasite for its growth and development. The possible iron sources are transferrin-bound iron (TBI) and non-transferrin bound iron (NTBI) in the plasma compartment [45–48], labile/transient iron [31] and ferritin iron [49] in red cell cytoplasm.

## Conclusions

GTE and CM1 could inhibit growth and development of *P. falciparum* culture, possibly by depriving the iron nutrient, which is essential for the fast-dividing malaria parasites. Predominantly, EGCG-enriched green tea would be beneficial for treatment of malaria-infected patients and amelioration of their haemolytic oxidative stress. Suggestively, green tea and CM1 may be used as therapeutic/preventive agents *per se* or in adjunction with PYR to increase efficacy of and eliminate resistance to anti-malarial drug. Prospectively, the effect of green tea on antioxidant defense mechanism and inflammation crisis of the patients needs to be further investigated.

## Abbreviations

ANOVA: one-way analysis of variance
CA-AM: calcein acetoxymethyl
CQ: chloroquine
DCF: oxidized dichlorofluorescein
DCFH: reduced dichlorofluorescein
DCFH-DA: 2′,7′-dichlorodihydrofluorescein diacetate
DFO: desferrioxamine
DFP: deferiprone
DFX: deferasirox
DHA: dihydroartemisinin
2,3-DHB: 2,3-dihydroxybenzoic acid
DHFR: dihydrofolate reductase
DHPS: dihydropteroate synthase
EGCG: epigallocatechin 3-gallate
EHPG: *N*,*N*′-ethylenebis(*o*-hydroxyphenylglycine)
FI: fluorescent intensity
GTE: green tea extract
HBED: *N*,*N′*-bis (*o*-hydroxybenzyl) ethylenediamine-*N*,*N′*-diacetic acid
HEPES: 4-(2-hydroxyethyl)-1-piperazine ethanesulfonic acid
IC_50_: half maximal inhibitory concentration
K_part_: partition coefficient
LIP: labile iron pool
LPI: labile plasma iron
NTBI: non-transferrin bound iron
PBS: phosphate buffered saline
pK_a_: equilibrium constant
PRBC: parasitized red blood cells
PYR: pyrimethamine
RBC: red blood cells
ROS: reactive oxygen species
S: sulfadoxine
